# Physical Therapy for Young Children Diagnosed with Autism Spectrum Disorders–Clinical Frameworks Model in an Israeli Setting

**DOI:** 10.3389/fped.2013.00019

**Published:** 2013-08-14

**Authors:** Osnat Atun-Einy, Meir Lotan, Yael Harel, Efrat Shavit, Shimshon Burstein, Gali Kempner

**Affiliations:** ^1^Physical Therapy Department, Faculty of Welfare Sciences and Health, Haifa University, Haifa, Israel; ^2^Physical Therapy Department, Faculty of Medicine Sciences, Ariel University, Ariel, Israel; ^3^Physical Therapy Services at the Association for Children at Risk, Tel-Aviv, Israel; ^4^Physical Therapy Services at Alut, The Israel National Autism Association, Alutaf, Givatayim, Israel; ^5^The Association for Children at Risk, Tel-Aviv, Israel

**Keywords:** physical therapy, intervention, ASD, autism, Israel

## Abstract

Recent research findings suggest that many children with Autism Spectrum Disorders (ASD) demonstrate delayed and atypical motor achievements. It has now become clear that a more holistic, integrative and multi-disciplinary intervention is required to effectively address the motor-related impairments of this population. It is also crucial to ensure that this group of clients has access to early physical therapy (PT) interventions. Despite accumulating research on physical interventions, little is known about intervention model for implementation at a national level. This report introduces a model that uniquely illustrates implementation of PT services for a large number of children with ASD. The model has been operating for the past 2 years in one country (Israel), and includes an optional implementation model of PT practice settings for young children diagnosed with ASD. The Israeli setting offers a unique opportunity for implementing PT services for a multitude of children with ASD on a regular basis as an accepted/needed service. The initial outcomes of the present implementation suggest that an intensive PT intervention program might enhance therapeutic outcomes for this population, and contribute to our knowledge on the potential of PT for individuals with ASD.

## Background

Autism Spectrum Disorder (ASD) is a neuro-developmental disorder characterized by impaired social interaction and communication, and by restricted and repetitive behavior (1). The clinical characteristics of ASD include fundamental deficits in social functioning and in language development and expression, and the presence of specific or repetitive interests and behaviors (2). The prevalence of ASD is constantly rising and was estimated last year by the Center for Disease Control (CDC) at 1 in 88 (3). The definition of ASD highlights the conventional clinical focus on the social, communicative and behavioral elements of the disorder, with little regard to physical involvement.

Autism predominately affects males, with a male-to-female ratio of approximately 4.3:1. (1). The extraordinary growth in the field of research and intervention in Autism in the past decade is related to the substantial increase in the number of children diagnosed with this disorder. As interest grew, and other elements involved in Autism were revealed, it became evident that many individuals with this disorder also show postural (4), motor (5), and functional delays (6). More specifically, previous reports suggest that many children with ASD demonstrate atypical motor development and delay in motor milestones achievements such as asymmetry, oral-motor problems, repetitive motor movements, dyspraxia (6), motor coordination (7), movement preparation reaction (8), and motor milestone delays (5). Recent studies suggest that movement disturbances play an intrinsic part in ASD (4, 9), are present from birth, and might even help early diagnosis of Autism in the first few months of life (10). Some of these difficulties present as delayed achievements on standardized motor assessments.

Ming et al. (11) described their findings from a cohort of 154 children with ASD. They found various motor aspects to be lacking in children with ASD, including hypotonia (51%), motor apraxia (34%), toe-walking (19%), and gross motor delays (9%). The presence of motoric challenges, regardless of whether they are primary or secondary to Autism, still has substantial implications for individualized educational interventions.

Motor delays and motor deficits which are mostly overlooked have been identified in children with ASD and might escalate with progressive age (12), regress into a set of chronic disorders, and could become increasingly pervasive with age (13). Therefore, it is crucial that this group of clients will take part in early intervention programs applied from the first few months of life (10). In order to understand the importance of PT physical intervention for this group of clients, there is a growing need to assess the effect of such programs.

## Motor Interventions for Individuals with ASD

Today there are few motor-oriented therapeutic interventions aimed at this group of clients, and articles researching this area are scarce.

In general, physical exercise is expected to reduce the risk of general health problems in individuals with ASD, as it does in the general population. Furthermore, several studies have reported a reducing effect of exercise on self-stimulatory behavior in individuals with ASD (14, 15).

Recent findings regarding the motor aspects of individuals with ASD suggest that a walking program improved the physical condition of adolescents with severe ASD and reduced their BMI index (16). Physical activity was found to have positive effects on social behavior (17), communication skills [Hameury et al., 2010 in (18)], academic engagement (19), and sensory skills (20). Despite the scarcity of research projects in this area, the cumulative evidence suggests that physical exercise improves the physical condition as well as other challenged realms of people with ASD.

Today, there is an understanding that motor skills are instrumental for learning skills in other areas (e.g., social behaviors, communication skills, academic engagement, and sensory skills) (6) and thus motor-related difficulties should be addressed as a core discipline within the educational curricula or through related therapy services during early childhood.

## Aims

The current report introduces a new model of physical therapy services for young children with ASD, which has been implemented in the field for the past 2 years and is designed to meet the complex needs of this group.

Exploring an ongoing implemented model has important theoretical and clinical implications. First – models of service delivery are crucial for future learning in the absence of an established model of PT service delivery for children with ASD [especially given that general recommendations for interventions with this population are based on best practice principles for examination and intervention with children with ASD and other developmental disabilities (21)].

Second – at the disciplinary level, models of service delivery facilitates outcomes research and evidence-based decision making (22).

Third, the proposed model might be implemented or challenged by other countries/organizations, with a future hope to enhance function and motor abilities of young individuals with ASD.

## The Israeli ASD Clinical Frameworks Model

The Israeli ASD Clinical Frameworks model proposes a new approach to PT services for children with ASD. It is important to describe the context in which this model was developed and the major categories of service delivery programs implemented in Israel.

### The Israeli context

In Israel, the incidence rate of ASD in 2008 was 1:214; according to official systems 2.442 children under the age of 7 receive support due to a diagnosis of ASD (23). In 2009, an amendment to Public Health Law 43 required an improvement in the service delivery to these clients and their families. The state acknowledged health care providers’ a key role in developing a comprehensive program for young children with ASD. In Israel there are about 50 day-care centers and communicational kindergartens for children with ASD at ages 1.5–7.5. The majority of all Israeli children diagnosed with ASD receive individualized educational and therapeutic services through two main associations that provide developmental, educational, and general healthcare services that fit the children’s needs: these associations are supervised by the Ministry of Health, Ministry of Education, and Ministry of Welfare and Social Services. Together, the facilities of these associations offer an array of therapeutic services that are implemented with 400 children by approximately 35 physical therapists on a regular basis. The proposed intervention model is designed to offer uniform (one assessment format for all) yet individualized (programs customized to each child’s needs and tendencies) assessment and therapeutic services for these young children diagnosed with ASD presenting a need for developmental enhancement.

### Themes and principles of the Israeli ASD Clinical Frameworks Model of PT service delivery

The Israeli ASD Clinical Frameworks model of service delivery incorporates several key themes:

(a) The intervention model is based on dynamic system theory, which emphasizes the interaction between factors arising from the individual, the environment, and the task (6).
(b) The intervention is aimed at enhancing function and exploratory behaviors in order to promote optimal independence and participation in everyday life.
(c) The intervention is implemented by focusing on the children’s strengths (e.g., their relative love for movement) to help them overcome their social and communicative impairments.

### Description of the Israeli ASD Clinical Frameworks Model

**Figure 1:**
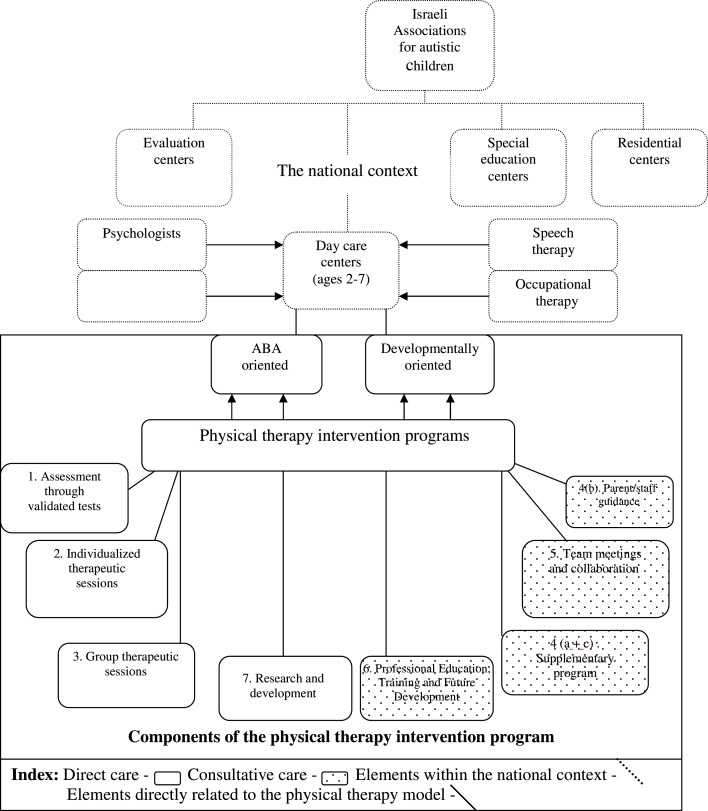
**A model of physical therapy services for children with ASD in Israel**.

The Israeli ASD Clinical Frameworks model integrates several key features, including assessment, design of intervention plans, and intervention layout, which are described below and in Figure 1). In general, each facility has one PT staff member. Approximately one-half of the children receive direct PT interventions according to the PT’s individual assessment, and approximately one-quarter of the second half are enrolled in specific goal-oriented group therapy that includes activities and skills such as playground activities, bicycle riding, balance and accuracy of ball playing.

## Establishing the Intervention Layout

The main goal of the physical therapy services is to improve the children’s participation and reduce their developmental and functional obstacles, in order to enable and encourage their inclusion in their respective peer groups. In general, most of the treatment activities are initiated in the therapy room, with the intention of exporting them to the child’s natural environment (the kindergarten space or playground) as soon as possible.

After an individual assessment by a physical therapist, an individualized intervention program is designed for each child. Program development entails the determination of: (a) program intensity; (b) program type of (hands-on, supplementary program, or both); (c) intervention modality (individual physical therapy intervention, group physical therapy intervention, supplementary therapeutic intervention implemented by the caregivers on a daily/weekly basis); (d) interdisciplinary coordination and staff meetings; and (e) research and development. The specific elements described by the model are elaborated in the following paragraphs.

### Assessment through validated tests

The child’s developmental functional and motor abilities are assessed individually at day-care centers (for children between the ages of 1.5 and 4 years) and in communication kindergartens (for children between the ages of 3 and 7.5 years). Assessment is based on (a) an anamnesis of the child’s medical records; (b) results of validated assessments tests, including international and Israeli standardized motor evaluations tests such as the Peabody Developmental Motor Scales (5), and Zuk Assessment for Motor Function and Movement Skills (24); (c) functional and daily living observations; and (d) questionnaires completed by caregivers and parents, such as Pediatric Evaluation of Disability Inventory, PEDI (25). A pre-intervention assessment is performed with each child upon his or her initial entry to the educational facility. Follow-up assessments are performed annually.

### Individualized therapeutic sessions

Practitioners incorporate strategies for enhancing the child’s joy of movement and movement initiation. These goals are incorporated with other developmental goals (including communicational, interaction, educational, A.D.L). The individualized intervention is designed to meet the specific needs of each individual with ASD, by organizing the treatment and its parts in a manner that reduces anxiety and encourages self-regulation (such as senso-motor regulation), which facilitates motor learning. The intervention program is focused on three main goals:

(a) Facilitate acquisition of lacking motor abilities in static as well as dynamic situations
(b) Facilitate acquisition of skills that enhance independent functioning in the peer group, family, and society
(c) Reduce the physical constraints presented by ASD

The program meets these goals through:

(a) Teaching tools that will assist the child in planning and organizing within his peer group, his family, and his society.
(b) Encouraging function-oriented movement rather than sensory or stereotypically oriented movement.
(c) Teaching tools that will assist the child cope with spatial orientation issues.
(d) Improving independence in daily situations.
(e) Improving posture in various daily situations.
(f) Improving movement patterns in different surroundings.
(g) Improving cardio-vascular abilities.
(h) Ergonomic adaptation of the child’s surroundings.
(i) Adaptation of assistive devices to reduce spatial insecurity and enhance spatial organization.
(j) Enhancement of educational and attentive abilities through sensory organization and centering (using sensory suits, weighted vests, trampolines).

Interventions are customized to each child’s abilities, anxiety level, and ability to accept changes, yet all sessions conform to a general fixed format. Intervention layout is based on regular, structured sessions that start with an opening ceremony (going to the therapy room, removing shoes, presenting the context of the intervention). The therapy session comprises a series of activities, beginning with activities of a more passive nature (sensory organizing, tone changing), proceeding to assisted active participation (arranging the room), and to a more active part of the session at a higher level of engagement (alignment, muscle strengthening, performing new functional tasks), ending within a functional context and a fixed, structured closing ceremony.

### Group therapeutic sessions

Group interventions can be implemented as direct interventions performed by the physical therapist or as a daily/weekly program implemented by the caregivers and supervised periodically by the physical therapist. Group sessions are conducted according to the needs of all children involved in the program, and in accordance with their performance level and the behavioral challenges they present. Group interventions can be implemented as a playground activity or as an indoor program.

(a) Enhancing the child’s motivation for movement through peer observation and imitation.
(b) Assisting the child with ASD in meeting basic demands of interaction with peers (taking turns, patience, acceptance of the needs and pace of others, acknowledging the wants of others).
(c) Assisting the child in acquiring imitational skills which composite a crucial part in learning and social acceptance and integration (26) for all children.
(d) Challenging the child to typical performance within regular daily surroundings such as playgrounds and different ball games.

Group interventions can be implemented in different modalities: (a) tailored PT treatment for individuals or pairs, with participation of another healthcare-related professional (such as occupational therapist or speech therapists); (b) PT treatment of two children instructed by PT, and two additional children who work with another staff member; (c) A group of up to four children, typically instructed by PT and another member of the caregiving staff. This option is designed as a model for the caregivers to operate on days the PT is not working in the facility.

### Parent/staff guidance and supervision

A key element in the proposed holistic program is supervision and guidance to caregivers, educational staff, parents, and other health care professionals, regarding the physical needs of each child with ASD and the ability to incorporate physical challenges as enhancers of appropriate educational and social behaviors. Supervision and guidance are provided through frontal lectures, presentation of intervention examples, and guidance booklets.

(a) Constructing and supervising programs to be implemented within the educational facility by the caregivers (playground activities, bicycle training, stair training).
(b) Constructing and supervising programs to be implemented at home or in the neighborhood surroundings by the parents. This could be done as periodical meetings within the educational facilities or through home based instructional meetings.
(c) Constructing and supervising motor intervention programs to be implemented with peers during the first attempts of integrating the child with ASD in the educational facility by the caregivers.

### Team meetings and collaboration

Interdisciplinary interactions with other health care professionals are conducted on a regular basis. At these meetings, members of the interdisciplinary staff jointly develop treatment goals, plan initiation of joint interventions, and share knowledge.

(a) Regular participation and involvement in routine educational facility and staff meetings on a weekly basis.
(b) Initiation and involvement in multi-disciplinary meetings in order to establish mutual goal setting and treatment program planning with regards to parental point of view.
(c) Regular participation and involvement in the assessment and intervention planning for specific children who present a complex array of co-morbidities.
(d) Attending joint home visitations with other staff members.
(e) Joint treatments with other healthcare professionals.

### Professional education, training, and future development

Working with children with ASD requires competent, experienced professionals with advanced knowledge and skills.

#### Entry level/professional education programs

Professional education for physical therapy students is offered through the educational programs at academic facilities (Physical therapy programs at Haifa and Ariel Universities).

#### Postgraduate level

Physical therapists working with this population are invited to workshops and conferences on ASD and receive supervision in regards to their professional experience and needs.

#### National level – the Autism Spectrum Disorders interest group

1. All leading clinical and academic physical therapists involved with this group of clients meet regularly on a monthly basis to discuss issues related to clinical care, and research and development of therapy plans.
2. Physical therapists working with this population are encouraged to become involved in case study presentation, clinical brain storming groups, workshops and conferences on the topic of ASD.
3. Novell physical therapists involved in direct interventions receive specific, individual instruction on a regular basis by experienced physical therapists, both as individual guidance meetings as well as group meetings.
4. Workshops and professional educational meetings for para-professionals from other disciplines working with individuals with ASD.
5. Emotional guidance and support to physical therapists working with children with ASD both on an individual and group level. These support groups are facilitated by experts who specialize in emotional therapies (psychologists, music therapists, art therapists).

### Research and development

A key competency in advancing care for any population should be knowledge gathering, and research and development. As PT is a relatively new as a core therapeutic discipline with children with ASD, it is of high importance to initiate some form of information gathering and outcome measurement collection. This information is later used as the base for Evidence Inspired Practice (EIP) with this population. Therefore, physical therapists working with children with ASD are involved at different levels of research with this group of clients. This involvement includes the initiation, evaluation, and reporting of specific case studies and intervention outcomes, data collection from large cohorts, and other related activities.

## Summary

The present article describes a novel an intensive model for implementing PT services for young children with ASD. As physical therapy for children with ASD is relatively new, this model may serve as a good starting point for future comparative studies that examine the efficacy and effectiveness of physical therapy intervention in Israel and elsewhere.

## Conflict of Interest Statement

The authors declare that the research was conducted in the absence of any commercial or financial relationships that could be construed as a potential conflict of interest.
